# C-Reactive Protein as a Predictor of Early Antibiotic Treatment Failure in Hospitalized Patients With Community-Acquired Pneumonia

**DOI:** 10.7759/cureus.111885

**Published:** 2026-07-01

**Authors:** Brayan Andres Yepiz Carrillo, Edgar Alejandro Garcia Robles, Rene Agustin Flores Franco

**Affiliations:** 1 Internal Medicine, Instituto Mexicano del Seguro Social, Hospital General Regional No. 1, Chihuahua, MEX; 2 Pulmonology, Instituto Mexicano del Seguro Social, Hospital General Regional No. 1, Chihuahua, MEX

**Keywords:** antibiotic treatment failure, biomarkers, clinical outcomes, community-acquired pneumonia, c-reactive protein, curb 65, hospitalized patients, inflammatory markers, retrospective study

## Abstract

Background: Community-acquired pneumonia (CAP) is one of the leading causes of hospitalization and infectious mortality worldwide. Identifying factors associated with early response to antibiotic treatment may improve clinical outcomes.

Methods: This retrospective, analytical, observational study included 230 adult patients hospitalized with CAP at Regional General Hospital No. 1 of the Mexican Social Security Institute in Chihuahua, Mexico, between June 2024 and December 2025. Clinical variables, comorbidities, inflammatory biomarkers, and severity scores were evaluated. Statistical analysis included chi-square tests and binary logistic regression.

Results: A total of 230/230 (100%) patients were included. The mean age was 63.9 ± 12.7 years, and 121/230 (52.6%) patients were male and 109/230 (47.4%) were female. A favorable response to the initial antibiotic regimen was observed in 143/230 (62.2%) patients, whereas 87/230 (37.8%) required therapeutic escalation. Mortality was higher among patients requiring escalation than those who did not (31/87 (35.6%) vs. 18/143 (12.6%), χ²=15.8, df=1, p<0.001, Cramér’s V=0.26). In multivariable analysis, higher baseline C-reactive protein (CRP) levels were independently associated with non-response to initial antibiotic therapy (OR=1.98; 95% CI: 1.10-3.55; p=0.022).

Conclusions: Higher baseline CRP levels were independently associated with non-response to the initial antibiotic regimen in patients with CAP.

## Introduction

Community-acquired pneumonia (CAP) is one of the most common diseases and remains a leading cause of hospitalization, with an estimated annual incidence of 12%. It is among the leading causes of death from infectious diseases globally, especially in patients with comorbidities [1,2].

In recent years, risk stratification in CAP has become increasingly important, as it has helped guide clinicians' initial therapeutic decisions, particularly in assessing the need for hospitalization and even antibiotic management. Historically, management has traditionally been based on comorbidities, severity, and clinical judgment, resulting in either underestimation or overestimation of the true severity of the disease [3-5].

To improve predictive models of severity and mortality and to determine whether to admit a patient to the hospital, the most prominent scales are CURB-65 and other severity scores, which are widely used [6-9]. Some studies suggest that the magnitude of the initial inflammatory response may also be associated with the early clinical course and the initial antimicrobial response, identifying clinical factors, comorbidities, biochemical markers, and initial prognostic scores [10-12].

The purpose of this study was to identify factors associated with response to the initial antibiotic regimen in patients hospitalized for CAP at Regional General Hospital No. 1 of the Mexican Social Security Institute (IMSS).

## Materials and methods

This was a retrospective, analytical, observational study of hospitalized patients diagnosed with CAP. The study was conducted in the Internal Medicine Department of Regional General Hospital No. 1 of the IMSS in Chihuahua, Mexico.

The information was obtained after protocol approval by the hospital’s local health research and ethics committee, through a review of the physical and electronic medical records of patients treated between June 2024 and December 2025. All patients who met the selection criteria were included: patients over 18 years of age hospitalized with a diagnosis of CAP who were receiving antimicrobial treatment from admission to the Internal Medicine Department. Initial empirical antimicrobial therapy was prescribed by the treating physicians according to institutional clinical practice, considering disease severity, patient comorbidities, and clinical judgment. Because of the retrospective nature of the study, no standardized antibiotic protocol was applied as part of the study design. Patients with criteria for hospital-acquired pneumonia, recent prior hospitalizations, significant immunosuppression, or incomplete records that precluded complete assessment of the study variables were excluded.

The sample size was estimated using a proportion formula, considering previous reports that described a non-response rate to initial antimicrobial treatment of approximately 15.9%. Based on this calculation, a sample of 207 patients was estimated; however, to account for an anticipated 10% of incomplete records, the target sample size was increased to 230 patients.

Clinical response was assessed within 48-72 hours using predefined clinical criteria obtained from the medical records. Response was defined as the absence of antibiotic escalation together with clinical stability or improvement, including temperature <37.8°C or decreasing, heart rate <100 beats/min, respiratory rate <24 breaths/min, systolic blood pressure >90 mmHg without vasopressor support, oxygen saturation ≥90% with stable or reduced oxygen requirements, and tolerance of oral intake. When available, a ≥50% reduction in C-reactive protein (CRP) by days 3-4 was also considered. Non-response was defined as the need for antibiotic escalation because of clinical deterioration, intensive care unit admission, mechanical ventilation, vasopressor requirement, death within 72 hours, or failure of CRP to decrease by at least 50% when follow-up measurements were available. All outcome variables were retrospectively extracted from routine medical records.

Independent variables included age, sex, and baseline CRP, which was analyzed as a continuous variable (mg/L). Clinical variables associated with the outcome in other cohorts were also analyzed, including diabetes, chronic lung disease, chronic kidney disease, and cardiovascular disease. Cardiovascular disease was defined as a documented history of hypertension, ischemic heart disease, heart failure, atrial fibrillation, valvular heart disease, or other chronic cardiovascular conditions recorded in the medical record. Lifestyle variables such as smoking and alcohol consumption were also considered. Additionally, severity scores such as CURB-65 were evaluated.

Data were collected using a standardized data collection form designed for this study and subsequently entered into an electronic database for analysis using Microsoft Excel (Microsoft Corp., Redmond, WA, USA) and SPSS (IBM Corp., Armonk, NY). Patients with incomplete records preventing assessment of the primary outcome were excluded. However, some secondary variables, including components required to calculate the CURB-65 score, were unavailable in a subset of patients because they were not consistently documented in the medical records. Quantitative variables were described using measures of central tendency and dispersion, whereas qualitative variables were expressed as frequencies and percentages.

Continuous variables were compared using Student’s t-test, while categorical variables were analyzed using Pearson’s chi-square test or Fisher’s exact test, as appropriate. Variables with p<0.10 in the bivariate analysis were considered for inclusion in the multivariable logistic regression model. The final model was constructed by considering both statistical significance and clinical relevance while taking into account the number of outcome events to minimize model overfitting.

## Results

A total of 230 patients hospitalized at Regional General Hospital No. 1 with a diagnosis of CAP were included in the study. The mean age was 63.9 ± 12.7 years (range, 22-80 years), and 121/230 (52.6%) patients were male. The baseline characteristics of the study population are summarized in Table 1. Comorbidities were common, particularly cardiovascular disease (164/230 (71.3%)), diabetes mellitus (146/230 (63.5%)), and smoking (156/230 (67.8%)).

**Table 1:** Baseline characteristics of the study population (N=230)

Variable	n (%)
Age, years (mean ± SD)	63.9 ± 12.7
Male sex, n (%)	121 (52.6%)
Female sex, n (%)	109 (47.4%)
Smoking	156 (67.8%)
Alcoholism	90 (39.1%)
Chronic lung disease	49 (21.3%)
Cardiovascular disease	164 (71.3%)
Diabetes mellitus	146 (63.5%)
Chronic kidney disease	20 (8.7%)

Associations between demographic, clinical, and biochemical variables and response to the initial antibiotic regimen are summarized in Table 2. No statistically significant differences were observed in sex distribution (male: 69/143 (47.9%) vs. 52/87 (59.8%), χ²=2.85, df=1, p=0.09, Cramér’s V=0.11). Similarly, smoking was not associated with response (94/143 (65.7%) vs. 62/87 (71.3%), χ²=0.61, df=1, p=0.43, Cramér’s V=0.05), nor was alcoholism (52/143 (36.4%) vs. 38/87 (43.7%), χ²=0.98, df=1, p=0.32, Cramér’s V=0.06). Chronic lung disease (36/143 (25.2%) vs. 13/87 (14.9%), χ²=2.70, df=1, p=0.10, Cramér’s V=0.11), chronic heart disease (108/143 (75.5%) vs. 56/87 (64.4%), χ²=2.52, df=1, p=0.11, Cramér’s V=0.10), diabetes mellitus (93/143 (65.0%) vs. 53/87 (60.9%), χ²=0.17, df=1, p=0.68, Cramér’s V=0.03), and chronic kidney disease (13/143 (9.1%) vs. 7/87 (8.0%), χ²=0.001, df=1, p=0.98, Cramér’s V=0.01) were also not significantly associated with response to treatment.

**Table 2:** Response to the initial antibiotic regimen (N=230) Data are presented as mean ± SD or n (%). Pearson’s chi-square test was used for categorical variables, and Student’s t-test was used for continuous variables. Effect size is reported as Cramér’s V for categorical variables. CRP, C-reactive protein.

Variable	Responders (n=143)	Non-responders (n=87)	Statistic	df	p-value	Effect size
Age, years (mean ± SD)	64.2 ± 12.7	63.3 ± 12.7	t=0.54	—	0.59	—
Male sex, n (%)	69 (47.9%)	52 (59.8%)	χ²=2.85	1	0.09	V=0.11
Smoking, n (%)	94 (65.7%)	62 (71.3%)	χ²=0.61	1	0.43	V=0.05
Alcoholism, n (%)	52 (36.4%)	38 (43.7%)	χ²=0.98	1	0.32	V=0.06
Chronic lung disease, n (%)	36 (25.2%)	13 (14.9%)	χ²=2.70	1	0.10	V=0.11
Chronic heart disease, n (%)	108 (75.5%)	56 (64.4%)	χ²=2.52	1	0.11	V=0.10
Diabetes mellitus, n (%)	93 (65.0%)	53 (60.9%)	χ²=0.17	1	0.68	V=0.03
Chronic kidney disease, n (%)	13 (9.1%)	7 (8.0%)	χ²=0.001	1	0.98	V=0.01
Baseline CRP, mg/L (mean ± SD)	115.0 ± 96.8	152.8 ± 118.8	t = -2.50	—	0.013	—

As shown in Table 3, 87/230 (37.8%) patients required escalation of the initial antibiotic regimen, while 143/230 (62.2%) did not require any modifications to their initial treatment. In the group requiring antibiotic escalation, 56/87 (64.4%) patients were discharged home and 31/87 (35.6%) died. In contrast, in the group that did not require escalation, 125/143 (87.4%) patients were discharged home and 18/143 (12.6%) died. A statistically significant association was observed between antibiotic escalation and clinical outcome, with a higher mortality rate in patients who required modification of the initial antibiotic treatment (χ²=15.8, df=1, p<0.001, Cramér’s V=0.26).

**Table 3:** Association between antibiotic escalation and clinical outcome in patients hospitalized for community-acquired pneumonia (N=230) Data are presented as n (%). Percentages are calculated by row. Pearson’s chi-square test was used. Effect size is reported as Cramér’s V.

Antibiotic escalation	Discharge home, n (%)	Death, n (%)	Statistic	df	p-value	Effect size
Yes (n=87)	56/87 (64.4%)	31/87 (35.6%)	χ²=15.8	1	<0.001	V=0.26
No (n=143)	125/143 (87.4%)	18/143 (12.6%)	—	—	—	—

CURB-65 could be calculated in 227/230 (98.7%) patients due to missing data in three cases. Of these, 30/227 (13.2%) had a score of 0-1; 29/30 (96.7%) were discharged, and 1/30 (3.3%) died. A total of 119/227 (52.4%) patients had a score of 2; of these, 102/119 (85.7%) were discharged, and 17/119 (14.3%) died. In patients with CURB-65 ≥3 (78/227 (34.4%)), 48/78 (61.5%) were discharged and 30/78 (38.5%) died. Mortality increased with higher CURB-65 scores, showing a significant association with clinical outcome (χ²=22.88, df=2, p<0.001, Cramér’s V=0.32) in Table 4.

**Table 4:** Association between CURB-65 score and clinical outcome (N=227)

CURB-65 score	Discharge home, n (%)	Death, n (%)	Statistic	df	p-value	Effect size
0-1	29/30 (96.7%)	1/30 (3.3%)	χ²=22.88	2	<0.001	V=0.32
2	102/119 (85.7%)	17/119 (14.3%)	—	—	—	—
≥3	48/78 (61.5%)	30/78 (38.5%)	—	—	—	—

Binary logistic regression was performed (Table 5), including variables that showed an association in the bivariate analysis (p<0.10). Higher baseline CRP levels were independently associated with non-response to the initial antibiotic regimen (OR=1.98; 95% CI: 1.10-3.55; p=0.022).

**Table 5:** Factors independently associated with non-response to the initial antibiotic regimen (binary logistic regression) CRP, C-reactive protein.

Variable	OR	95% CI	p-value
Male sex	1.21	0.67-2.18	0.53
Chronic lung disease	0.49	0.23-1.05	0.065
Baseline CRP (mg/L)	1.98	1.10-3.55	0.022

## Discussion

In this study, it was observed that hospitalized patients with higher baseline CRP levels upon admission showed a higher frequency of non-response to the initial antimicrobial regimen than those with lower values, suggesting that a more intense inflammatory response upon admission could be related to an unfavorable course during the first 72 hours of treatment, resulting in worse outcomes.

The cohort included 230 patients hospitalized for CAP who received antibiotic therapy. A total of 87/230 (37.8%) patients did not respond to the initial antibiotic treatment, resulting in a worse outcome [9]. This is consistent with previous studies reporting that early treatment failure can occur in nearly one-third of patients with CAP, reinforcing the fact that the initial course of patients with CAP can be unpredictable even when initial empirical treatment is administered according to standard recommendations established by international guidelines [4,13,14].

The observed non-response rate (37.8%) was higher than the 15.9% rate used for the sample size calculation. This discrepancy likely reflects differences between our study population and the reference cohort. Our study included only hospitalized patients, many of whom had a high burden of comorbidities and moderate-to-severe disease, which may have increased the likelihood of early treatment failure. In addition, our operational definition of non-response, based on antibiotic escalation or documented clinical deterioration during hospitalization, may not have been identical to that used in the reference study. Therefore, the higher observed non-response rate is more likely attributable to differences in patient characteristics and outcome definitions than to an error in the original sample size estimation.

In the bivariate analysis, patients with higher baseline CRP levels had a higher frequency of treatment failure. This association remained significant in the multivariable analysis, where higher baseline CRP levels were independently associated with failure of the initial antibiotic regimen (OR=1.98; 95% CI: 1.10-3.55; p=0.022). These findings suggest that the magnitude of the inflammatory response at hospital admission may be associated with the early clinical course of patients with CAP and could support closer clinical monitoring [10-15].

In studies evaluating acute-phase reactants as inflammatory biomarkers in this disease, CRP, unlike procalcitonin, does not differentiate the causative agent. However, both continue to be used primarily to assess treatment response and guide treatment duration. Nevertheless, when used as early predictors, the results remain debatable [10,14,15]. While CRP can be elevated by multiple comorbidities, it could guide clinicians when other more specific biomarkers are unavailable. Our findings suggest that higher baseline CRP levels are associated with an increased likelihood of non-response to the initial antibiotic regimen. However, because of the retrospective observational design, this study cannot determine whether the use of CRP to guide clinical management improves patient outcomes. Prospective studies are needed to evaluate its clinical utility as a decision-making tool.

Other clinical variables traditionally associated with worse outcomes in CAP, including diabetes, chronic lung disease, and cardiovascular disease, were not significantly associated with failure of the initial antibiotic regimen in our cohort. These findings should be interpreted cautiously and should not be considered evidence of the absence of an association. The lack of statistical significance may reflect the relatively homogeneous population of hospitalized patients, limited statistical power to detect modest effects, or residual confounding related to the retrospective design. Notably, chronic lung disease showed a trend toward an association in the multivariable analysis (OR=0.49; 95% CI: 0.23-1.05), suggesting that a clinically relevant effect cannot be excluded. Larger prospective studies are needed to clarify the contribution of these comorbidities to early treatment failure.

Among the most relevant findings was the mortality rate in patients who required escalation of the initial antimicrobial regimen. This could reflect that a lack of early response to antimicrobial treatment is more common in patients with more severe disease or a higher inflammatory burden. Early therapeutic failure has also been associated with longer hospital stays, a greater need for ventilatory support, and higher mortality [5,16-19]. The relatively high overall mortality observed in our cohort should also be interpreted in the context of the study setting. As this study was conducted at a tertiary referral hospital, the population consisted exclusively of hospitalized patients, many with multiple comorbidities and more severe illness than those managed in the outpatient setting. In addition, unmeasured factors such as delays in presentation, referral patterns, or access to healthcare may have influenced patient outcomes, although these variables were not evaluated in the present study. Therefore, the early identification of patients at risk of an unfavorable outcome remains a primary objective in the clinical management of CAP.

This study has several limitations that should be considered. First, its retrospective design, based on medical record review, may have affected the completeness and quality of the collected data. Incomplete documentation of some clinical variables, particularly those required to calculate severity scores such as CURB-65, resulted in missing data and limited the analysis of these variables. Furthermore, the study was conducted at a single tertiary-care hospital, which may limit the generalizability of the findings to other settings.

Additionally, serial CRP measurements were available in fewer than half of the patients, precluding a reliable assessment of CRP kinetics over time. Because changes in CRP during the first 48-72 hours have been reported to provide additional prognostic information beyond a single baseline measurement, the absence of standardized serial measurements represents an important limitation of this study. Consequently, our findings should be interpreted as demonstrating an association between baseline CRP and non-response to the initial antibiotic regimen rather than establishing the predictive value of CRP kinetics. Prospective studies with standardized serial CRP assessment are needed to determine whether changes in CRP improve risk stratification and clinical decision-making.

Although predefined objective criteria were used to classify treatment response, these variables were retrospectively extracted from routine medical records. Therefore, variability in clinical documentation among treating physicians may have resulted in some degree of outcome misclassification, potentially affecting the estimated strength of the association between baseline CRP and treatment response.

Another important limitation is the heterogeneity of the empirical antimicrobial regimens used. Initial antibiotic therapy was selected according to the treating physician’s clinical judgment, disease severity, patient comorbidities, and institutional practice rather than a standardized study protocol. Because microbiological cultures were not routinely performed, our study could not determine whether treatment failure resulted from inappropriate empirical antimicrobial therapy, infection with resistant pathogens, inadequate coverage of atypical organisms, or differences in the host inflammatory response. Therefore, residual confounding related to antimicrobial selection cannot be excluded, and the observed association between baseline CRP and treatment response should be interpreted within this context. In addition, although the diagnosis of CAP was established by the treating physicians based on compatible clinical and radiographic findings documented in the medical records, overlap with decompensated heart failure cannot be completely excluded because of the retrospective design, and some degree of diagnostic misclassification is therefore possible.

Despite these limitations, our findings support an association between higher baseline CRP levels and non-response to the initial antibiotic regimen. Whether incorporating baseline CRP into clinical decision-making improves patient outcomes remains uncertain and should be evaluated in prospective studies.

## Conclusions

Higher baseline CRP levels were associated with non-response to the initial antibiotic regimen in patients with CAP. These findings support an association but do not establish CRP as a clinically actionable predictive tool. Prospective studies are required to determine whether incorporating CRP into clinical management improves patient outcomes.
